# Characterization of the complete chloroplast genome of *Aesculus pavia*

**DOI:** 10.1080/23802359.2026.2619291

**Published:** 2026-01-22

**Authors:** Huihui Lin, Xiangxiao Meng, Huihua Wan, Xiuhong Mao, Wei Sun, Weiqiang Chen, Xuehua Xie

**Affiliations:** a State Key Laboratory for Quality Ensurance and Sustainable Use of Dao-di Herbs, Institute of Chinese Materia Medica, China Academy of Chinese Medical Sciences, Beijing, China; b Faculty of Medicinal Plants and Pharmacognosy, School of Traditional Chinese Medicine, Southern Medical University, Guangzhou, Guangdong Province, China; c Forest and Grass Seed Industry Research Institute, Shandong Academy of Forestry, Jinan, Shandong Province, China

**Keywords:** *Aesculus pavia*, chloroplast genome, phylogenetic relationship

## Abstract

*Aesculus pavia* L., a member of the genus *Aesculus* in the family Sapindaceae, holds significant value as both a medicinal and ornamental plant. In this study, we assembled and annotated the complete chloroplast genome of *A. pavia* and conducted the phylogenetic analysis among the genus *Aesculus*. The complete chloroplast genome of *A. pavia* is 156,394 bp in length, with a GC content of 37.90%. It exhibits a typical quadripartite structure, consisting of a large single-copy (LSC) region (85,927 bp), a small single-copy (SSC) region (18,751 bp), and a pair of inverted repeats (IRs) regions (25,858 bp). A total of 133 genes were annotated, including 88 protein-coding genes (PCG), 37 tRNA genes, and 8 rRNA genes. Phylogenetic analyses revealed that *A. pavia* is clustered with *A. turbinata* and *A. hippocastanum*, suggesting a close relationship between *A. pavia* and the two species. This study provides a new plastome sequence for evolutionary and phylogenetic studies of the genus *Aesculus*.

## 1. Introduction

The genus *Aesculus* comprises over 30 species distributed across Asia, Europe, and America, which are widely cultivated as ornamental trees and utilized as woody medicinal plants. *Aesculus pavia* L. (1753), a shrubby or small tree species characterized by striking red flowers, is primarily distributed in eastern Asia, eastern North America, and Europe (Little 1980). *A. pavia* is an important hybrid parent; its hybrid offspring, such as “Briotii,” “Neill Red,” exhibit high ornamental value and stress resistance. Ecologically, *A. pavia* exerts multiple pivotal functions in forest ecosystems, such as supporting early-spring pollinators, supplying food for rodents, and preventing soil erosion, and so on. The seeds of *A. pavia* have been utilized as an astringent to treat diarrhea, hemorrhoids, chronic venous insufficiency, and post-operative edema (Sirtori 2001). Studies on *A. pavia* focused on its bioactive compounds and pharmacological uses (Sun et al. 2011; Zhang and Li 2007; Zhang et al. 2006).

Molecular genetic resources are essential for species purity detection, as well as taxonomic, conservation, ecological, and evolutionary research. The chloroplast genome is an important molecular resource for species identification and phylogenetic analysis (Guo et al. 2023). Although chloroplast genome studies have been conducted on several species within the genus *Aesculus* (Liu et al. 2020; Zhang et al. 2019; Zheng et al. 2018), these efforts remain far from sufficient for an in-depth investigation into the phylogenetic relationships of the genus. For instance, partial chloroplast DNA markers (*matK*, *trnD*-*trnT*, *trnH*-*trnK*, *rps16*) of *A. sylvatica* and *A. flava* were analyzed to evaluate the contribution of historical contact, hybridization, and phylogeography (Modliszewski et al. 2006). Additionally, the phylogeny of the tribe Hippocastaneae (Sapindaceae) and comparative analyses were conducted using RAD-seq data to gain insights into the evolution and biogeography of the group (Du et al. 2020). Despite these advances, the complete chloroplast genome sequence of *A. pavia* remains unreported to date, which limits our ability to resolve fine-scale phylogenetic relationships, uncover chloroplast genome structural variations, and identify high-resolution molecular markers for population genetics research.

In this study, we assembled and annotated the chloroplast genome of *A. pavia* for the first time. Furthermore, we investigated its phylogenetic relationships within the genus *Aesculus* based on whole chloroplast genome sequences. The results of this study provide valuable data support for further exploring the evolutionary history, species conservation, and taxonomic classification of the genus *Aesculus*.

## 2. Materials and methods

### 2.1. Plant material, DNA extraction, and sequencing

**Figure 1.**
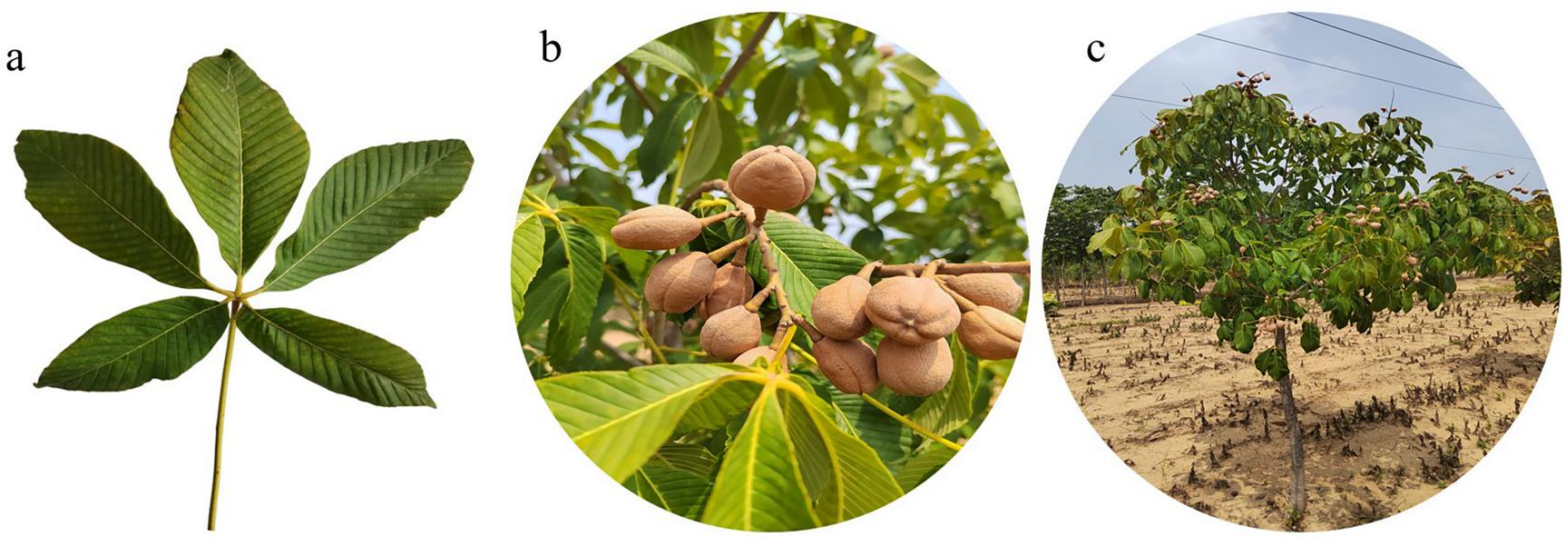
The morphological characteristics of *A. pavia.* (a) The leaves of *A. pavia*. palmate compound leaves, usually, there are 5 leaflets, and 5–7 leaflets on mature trees. The leaf blades are 7.5–15 centimeters long, green in summer and golden in autumn. (b) The seeds of *A. pavia.* The fruits are spherical, 2.5–5 centimeters in diameter, light brown. There are 1–3 seeds in each fruit, and the seeds are poisonous. (c) Plant individuals of *A. pavia.* The tree is 5–8 meters tall, with grayish - brown bark. The photos were taken by Xiuhong Mao in Jinan County, Shandong Province, China.

Fresh leaves of *A. pavia* were collected from Jinan, Shandong Province, China (Figure 1, 36°40′N, 117°00′E) by Dr. XiuHong Mao, and subsequently desiccated using silica gel. The voucher specimen was identified by XiuHong Mao and deposited in the National Traditional Chinese Medicine GeneBank of the Institute of Chinese Materia Medica, China Academy of Chinese Medical Sciences (Voucher number QYS20250824, Xuehua Xie, xie513812@163.com).

Total genomic DNA was extracted from leaf samples using the Plant Genomic DNA Kit (Tiangen, Beijing, China). DNA quantity was determined using Qubit^®^ 3.0 Fluorometer (Life Technologies, CA, USA). DNA sequencing was carried out using the DNBSEQ-T7 with 150 bp paired-end (MGI, China). Library construction and sequencing were performed by Annoroad Gene Technology Co., Ltd (Beijing, China). The raw data were filtered using fastp v0.26.0 (Chen 2025) with default parameters, yielding 7.71 GB of clean reads.

### 2.2. Chloroplast genome assembly and annotation

After quality control, the clean reads were used to assemble the chloroplast genome by using GetOrganelle v1.7.7.1 (Jin et al. 2020) with the parameter “-R 10-t 1-k 75,85,95,105-F embplant_pt.” The chloroplast genome of *A. hippocastanum* (NC_066015) which is closely related in terms of phylogenetic relationship was used as the reference (Du et al. 2020). The assembled scaffolds and their connectivity were visualized and adjusted by using Bandage v0.8.1. The annotation was carried out using the default parameters of CPGAVAS2 (Shi et al. 2019) (http://47.96.249.172:16019/analyzer/home), with *A. hippocastanum* (NC_066015) as the reference. The annotation results were manually refined by comparing with the reference genome using CPStools v2.5 (Huang et al. 2024) and rechecked using GESEQ (https://chlorobox.mpimp-golm.mpg.de/geseq.html) (Tillich et al. 2017) to generate the final annotated file. The chloroplast genome structure was visualized using CPGView (http://www.1kmpg.cn/cpgview) (Liu et al. 2023). The annotated chloroplast genome sequence was deposited in GenBank under the accession number PX251891. Simple sequence repeats (SSRs) in the chloroplast genome were identified using Misa (https://webblast.ipk-gatersleben.de/misa/) (Beier et al. 2017) and the SSRs sub-commands of Cpstools v2.5 (Huang et al. 2024).

### 2.3. Phylogenetic analysis

To investigate the evolutionary relationships of *A. pavia* within *Aeculus*, we downloaded 10 chloroplast genome sequences of *Aesculus* and 2 chloroplast genome sequences of *Acer* species from the NCBI database (http://www.ncbi.nlm.nih.gov/). Among them, *Acer saccharum* and *Acer rubrum* were selected as the outgroup. The 12 chloroplast genome sequences were aligned using MAFFT v7.310 (Rozewicki et al. 2019) with default parameters. Following sequence alignment, the resulting datasets were trimmed using TrimAl v1.5.0 (Capella-Gutiérrez et al. 2009) with the specified “-automated1” parameter. Phylogenetic analysis was conducted in IQ-TREE v2.4.0 (Minh et al. 2020) *via* the maximum-likelihood (ML) method with 1000 bootstrap replicates, based on the optimal substitution model (TVM + F + I + G4) selected under the Akaike Information Criterion (AIC) using the software’s built-in ModelFinder module (Kalyaanamoorthy et al. 2017). The resulting phylogenetic tree was visualized using the iTOL v7.2.1 web server (Letunic and Bork 2021).

## 3. Results

### 3.1. Characteristics of the chloroplast genome

**Figure 2.**
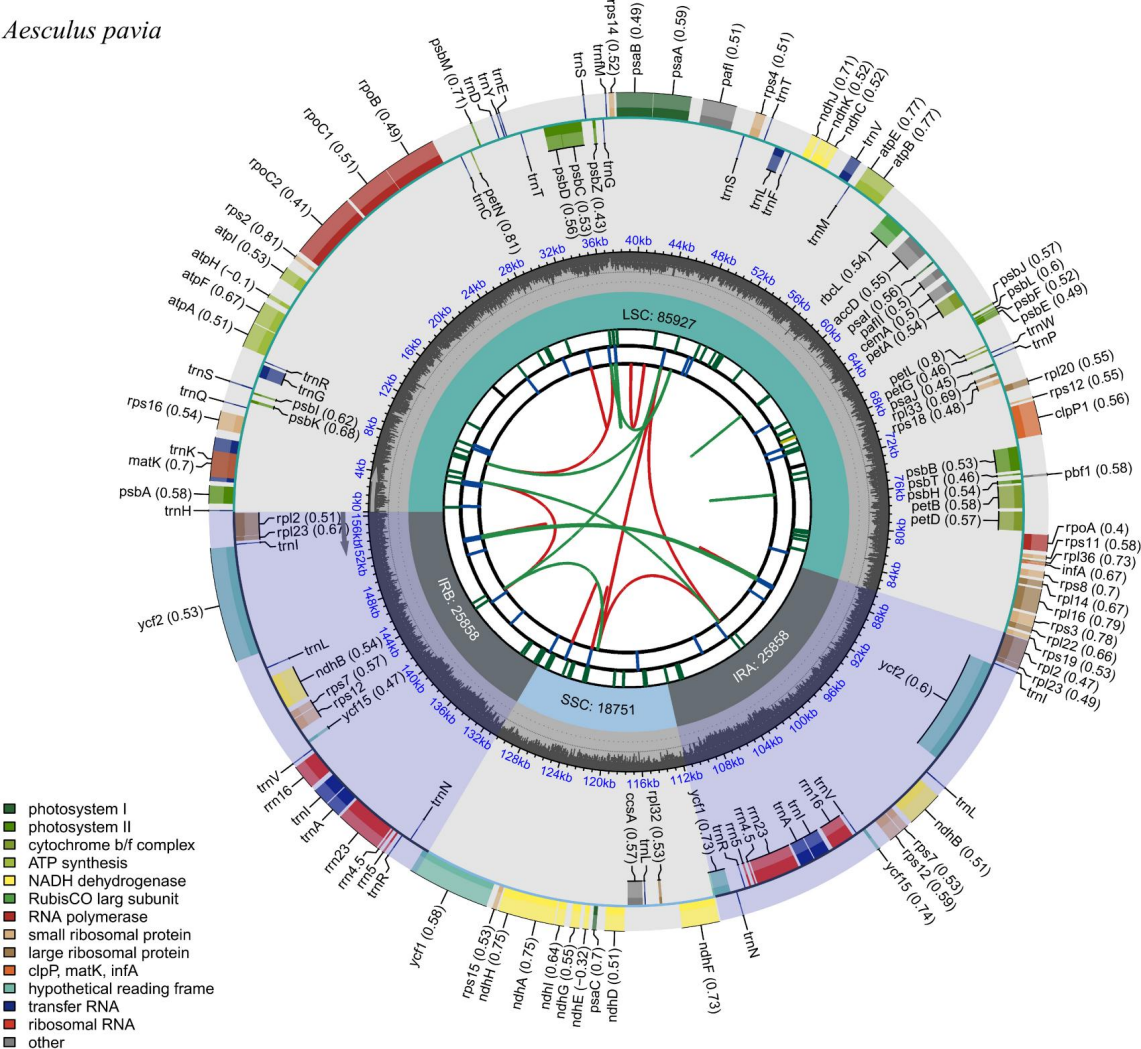
Gene map representing the chloroplast genome of *A. pavia*. As shown, the figure is consisting six of circles from the center to the outside, the innermost circle shows the forward and reverse repeats connected with the red and green arcs, respectively. The second circle and the third circle show the long tandem repeats and short tandem repeats or microsatellite sequences marked with short strips, respectively. The fourth circle exhibits the locations and length of the large single-copy (LSC) regions, small single-copy (SSC) and inverted repeat (IRA and IRB) regions. The fifth and sixth circle display GC content and the genes’ function categories as shown in different colors. The genes outside the outermost circle are transcribed anticlockwise, while the genes inside are transcribed clockwise. The number in parenthesis after gene name indicates codon usage bias.

The chloroplast complete genome of *A. pavia* exhibited a typical quadripartite structure, with a total length of 156,394 bp and a GC content of 37.90% (Figure 2). It consisted of a large single-copy (LSC) region of 85,927 bp, a small single-copy (SSC) region of 18,751 bp, and a pair of inverted repeat regions (IRa and IRb), each 25,858 bp in length (Figure 2). The chloroplast genome was sequenced with an average coverage of 4473× (range: 1195×–8596×), which effectively minimized random sequencing errors through consensus calling. More than 95% of the genome regions had a coverage ≥50×, ensuring high confidence in base calling (Figure S1).

A total of 133 genes were annotated in the chloroplast genome of *A. pavia*, including 88 PCGs, 37 transfer RNAs, and 8 ribosomal RNAs. The LSC region contained 69 PCGs and 22 tRNAs. The SSC region contained 12 PCGs and one tRNA. Within the IR regions. Seven PCGs, all rRNAs and seven tRNAs were duplicated. Among these, 10 PCGs (*rps16*, *atpF*, *rpoC1*, *petB*, *petD*, *rpl16*, *rpl2*, *ycf2*, *ndhB*, *ndhA*) contain an intron, and three PCGs (*pafI*, *clpP1*, *ycf1*) contain two introns (Figure S2). All 16 of these genes are cis-splicing genes, which play crucial roles in ensuring the integrity of ribosome biogenesis and protein translation, directly affecting photosynthetic efficiency and plant growth and development (Huo et al. 2024; Wang et al. 2022). Additionally, the rps12 gene was identified as a trans-splicing gene with three exons (Figure S3), which is essential for the stable operation of basic functions such as photosynthetic systems, energy metabolism, and ribosome assembly (Lee et al. 2019).

We compared and mapped whole chloroplast gene alignments among 10 *Aesculus* species using mVISTA, using the published cp genome of *A. assamica* (NC_056237) as the reference. The results showed that the divergence in the coding regions was greater than that in the noncoding regions (Figure S4). SSRs were analyzed using Misa and CPStools, and the results generated by these two analytical tools were consistent. A total of 71 SSRs were detected in *A. pavia* chloroplast genome, and with mononucleotide repeats being the most abundant (70 out of 71 SSRs).

### 3.2. Phylogenetic relationship

**Figure 3.**
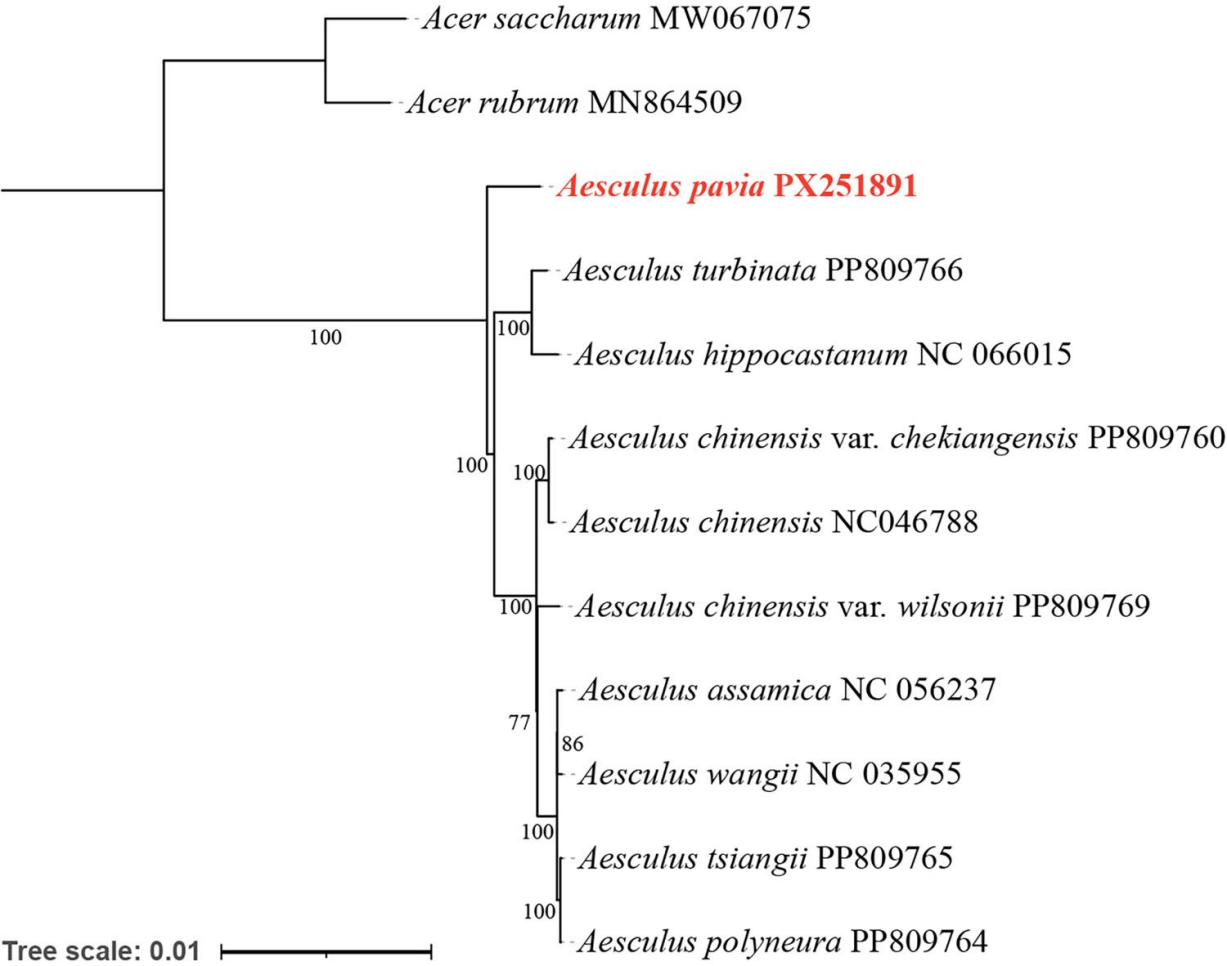
Maximum-likelihood (ML) phylogenetic tree based on the complete chloroplast genome sequence of 11 species from the sapindaceae. Numbers at each node correspond to bootstrap values calculated from 1,000 repetitions. The chloroplast genomes of *A. pavia* in this study were labeled in red and marked with a red star. The sequences used for constructing the phylogenetic tree are as follows: *Acer saccharum* MW067075 (unpublished), *acer rubrum* MN864509 (unpublished), *A. turbinata* PP809766 (unpublished), *A. hippocastanum* NC_066015 (unpublished), *A. chinensis* var. *chekiangensis* PP809760 (unpublished); *A. chinensis* NC_046788 (Zhang et al.^2019^); *A. chinensis* var. *wilsonii* PP809769 (unpublished); *A. assamica* NC_056237 (unpublished); *A. wangii* NC_035955 (Zheng et al. 2018); *A. tsiangii* PP809765 (unpublished); *A. polyneura* PP809764 (unpublished).

Phylogenetic analysis was performed using 10 chloroplast genomes from *Aesculus* species, *Acer saccharum,* and *Acer rubrum* as outgroup. The ML phylogenetic tree based on the 12 cp genomes revealed two major clades within the genus *Aesculus* (Figure 3), largely consistent with previous studies (Du et al. 2020). *A. pavia* was closely related to *A. turbinata* and *A. hippocastanum*. Most nodes in the tree exhibited a bootstrap support value of 100%, while only a few nodes in the terminal branches showed slightly lower support values. This indicates that the branching relationships of this phylogenetic tree are generally highly reliable.

## 4. Discussion and conclusions

In this study, the complete chloroplast genome of *A. pavia* was reported for the first time, containing 133 genes, including 88 PCGs, 37 tRNA genes, and 8 rRNA genes. The chloroplast genome of *A. pavia* is similar in size and structure to those of other reported *Aesculus* species, which indicates a relatively conserved chloroplast genome in this genus. We quantified the gene content of the chloroplast genomes from two closely related species, *A. hippocastanum* and *A. turbinata*, and the results demonstrated that the total number of genes was consistent across all three species (Table S1).

Comparative analysis of the inverted repeat (IR) boundaries revealed that the IR region boundaries of *A. pavia* and closely related species exhibited varying degrees of expansion and contraction (Figure S4). In particular, Notably, the lengths of the intergenic spacer regions at the JSB boundary (the junction of IRb and SSC) and the JSA boundary (the junction of IRa and SSC) exhibited distinct expansion. This is one of the core factors contributing to the slight divergence in their total genome lengths (Saina et al. 2018) and also reflects the evolutionary diversity of chloroplast genomes within the genus *Aesculus*.

Phylogenetic analysis showed that *A. pavia* was closely related to *A. turbinata* and *A. hippocastanum*. This finding is consistent with previous studies based on RAD sequencing data (Du et al. 2020). The cp genome sequence of *A. pavia* determined in this study provides important information for phylogenetic and evolutionary studies in *Aesculus*.

## Supplementary Material

03Supplementary Material for review20260106.doc

## Data Availability

The genome sequence data that support the findings of this study are openly available in GenBank of NCBI at https://www.ncbi.nlm.nih.gov under the accession number PX251891. The associated BioProject, SRA, and Bio-Sample numbers are PRJNA1320835, SRR35276515, and SAMN51175175, respectively.
