# Supplementary material for: Characterization of the complete chloroplast genome of Aesculus pavia
Source: Mitochondrial DNA B Resour. 2026 Jan 22;11(2):280–4. doi: 10.1080/23802359.2026.2619291 (PMC12829419; doi:10.1080/23802359.2026.2619291)
Supplement: 03Supplementary Material for review20260106.doc [file TMDN_A_2619291_SM4650.doc]

**Characterization of the complete chloroplast genome of *Aesculus pavia***

Huihui Lin^a,b^, Xiangxiao Meng^a^, Huihua Wan^a^, Xiuhong Mao^c^, Wei Sun^a^, Weiqiang Chen^a^, Xuehua Xie^a^

a State Key Laboratory for Quality Ensurance and Sustainable Use of Dao-di Herbs, Institute of Chinese Materia Medica, China Academy of Chinese Medical Sciences, Beijing, China

b Faculty of Medicinal Plants and Pharmacognosy, School of Traditional Chinese Medicine, Southern Medical University, Guangzhou, Guangdong province, China.

c Institute of ornamental plants, Shandong Academy of Forestry, Jinan, Shandong province, China.

CONTACT Xuehua Xie xie513812@163.com State Key Laboratory for Quality Ensurance and Sustainable Use of Dao-di Herbs, Institute of Chinese Materia Medica, China Academy of Chinese Medical Sciences, Beijing, China

**ABSTRACT**

*Aesculus pavia* L., a member of the genus *Aesculus* in the family Sapindaceae, holds significant value as both a medicinal and ornamental plant. In this study, we assembled and annotated the complete chloroplast genome of *A. pavia* and conducted the phylogenetic analysis among the genus *Aesculus*. The complete chloroplast genome of *A. pavia* is 156,394 bp in length, with a GC content of 37.90%. It exhibits a typical quadripartite structure, consisting of a large single-copy (LSC) region (85,927 bp), a small single-copy (SSC) region (18,751 bp), and a pair of inverted repeats (IRs) regions (25,858 bp). A total of 133 genes were annotated, including 88 protein-coding genes (PCG), 37 tRNA genes, and 8 rRNA genes. Phylogenetic analyses revealed that *A. pavia* is clustered with *A. turbinata* and *A. hippocastanum*, suggesting a close relationship between *A. pavia* and the two species. This study provides a new plastome sequence for evolutionary and phylogenetic studies of genus *Aesculus*.

**KEY WORDS:** *Aesculus pavia*; chloroplast genome; phylogenetic relationship

1. **Introduction**

The genus *Aesculus* comprises over 30 species distributed across Asia, Europe, and America, which are widely cultivated as ornamental trees and utilized as woody medicinal plants. *Aesculus pavia* L. (1753), a shrubby or small tree species characterized by striking red flowers, is primarily distributed in eastern Asia, eastern North America, and Europe (Little, 1980). *A. pavia* is an important hybrid parent, its hybrid offspring such as ‘Briotii’, ‘Neill Red’, exhibit high ornamental value and stress resistance. Ecologically, *A. pavia* exerts multiple pivotal functions in forest ecosystems, such as supporting early-spring pollinators, supplying food for rodents, and preventing soil erosion, and so on. The seeds of *A. pavia* have been utilized as an astringent to treat diarrhea, hemorrhoids, chronic venous insufficiency, and post-operative edema (Sirtori, C R,2001). Studies on *A. pavia* focused on its bioactive compounds and pharmacological uses (Sun, Z, et al.,2011; Zhang, Z and Li, S,2007; Zhang, Z, et al.,2006).

Molecular genetic resources are essential for species purity detection, as well as taxonomic, conservation, ecological, and evolutionary research. The chloroplast genome is an important molecular resource for species identification and phylogenetic analysis (Guo, C, et al.,2023). Although chloroplast genome studies have been conducted on several species within the genus *Aesculus* (Liu, Z, et al.,2020; Zhang, Z, et al.,2019; Zheng, W, et al.,2018), these efforts remain far from sufficient for an in-depth investigation into the phylogenetic relationships of the genus. For instance, partial chloroplast DNA markers (*matK*, *trnD*-*trnT*, *trnH*-*trnK*, *rps16*) of *A. sylvatica* and *A. flava* were analyzed to evaluate the contribution of historical contact, hybridization, and phylogeography (Modliszewski, J L, et al.,2006). Additionally, the phylogeny of the tribe Hippocastaneae (Sapindaceae) and comparative analyses were conducted using RAD-seq data to gain insights into the evolution and biogeography of the group (Du, Z, et al.,2020). Despite these advances, the complete chloroplast genome sequence of *A. pavia* remains unreported to date, which limits our ability to resolve fine-scale phylogenetic relationships, uncover chloroplast genome structural variations, and identify high-resolution molecular markers for population genetics research.

In this study, we assembled and annotated the chloroplast genome of *A. pavia* for the first time. Furthermore, we investigated its phylogenetic relationships within the genus *Aesculus* based on whole chloroplast genome sequences. The results of this study provide valuable data support for further exploring the evolutionary history, species conservation, and taxonomic classification of the genus *Aesculus*.

1. **Materials and methods**

2.1 Plant material, DNA extraction, and sequencing

Fresh leaves of *A. pavia* were collected from Jinan, Shandong Province, China (Figure 1, 36°40′N, 117°00′E) by Dr. XiuHong Mao, and subsequently desiccated using silica gel. The voucher specimen was identified by XiuHong Mao and deposited in the National Traditional Chinese Medicine GeneBank of the Institute of Chinese Materia Medica, China Academy of Chinese Medical Sciences (Voucher number QYS20250824, Xuehua Xie, xie513812@163.com).

Total genomic DNA was extracted from leaf samples using the Plant Genomic DNA Kit (Tiangen, Beijing, China). DNA quantity was determined using Qubit® 3.0 Fluorometer (Life Technologies, CA, USA). DNA sequencing was carried out using the DNBSEQ-T7 with 150 bp paired-end (MGI, China), Library construction and sequencing were performed by Annoroad Gene Technology Co., Ltd (Beijing, China). The raw data were filtered using fastp v0.26.0 (Chen, S,2025) with default parameters, yielding 7.71 GB of clean reads.

2.2 Chloroplast genome assembly and annotation

After quality control, the clean reads were used to assemble the chloroplast genome by using GetOrganelle v1.7.7.1 (Jin, J, et al.,2020) with the parameter “-R 10 -t 1 -k 75,85,95,105 -F embplant_pt”. The chloroplast genome of *A*. *hippocastanum* (NC_066015) which is closely related in terms of phylogenetic relationship was used as the reference (Du, Z, et al.,2020). The assembled scaffolds and their connectivity were visualized and adjusted by using Bandage v0.8.1. The annotation was carried out using the default parameters of CPGAVAS2 (Shi, L, et al.,2019) (http://47.96.249.172:16019/analyzer/home), with *A*. *hippocastanum* (NC_066015) as the reference. The annotation results were manually refined by comparing with the reference genome using CPStools v2.5 (Huang, L, et al.,2024) and rechecked using GESEQ (https://chlorobox.mpimp-golm.mpg.de/geseq.html) (Tillich, M, et al.,2017) to generate the final annotated file. The chloroplast genome structure was visualized using CPGView (<http://www.1kmpg.cn/cpgview>) (Liu, S, et al.,2023). The annotated chloroplast genome sequence was deposited in GenBank under the accession number PX251891. Simple sequence repeats (SSRs) in the chloroplast genome were identified using Misa (https://webblast.ipk-gatersleben.de/misa/) (Beier, S, et al.,2017) and the SSRs sub-commands of Cpstools v2.5 (Huang, L, et al.,2024).

2.3 Phylogenetic Analysis

To investigate the evolutionary relationships of *A. pavia* within *Aeculus*, we downloaded 10 chloroplast genome sequences of *Aesculus* and 2 chloroplast genome sequences of *Acer* species from the NCBI database (http://www.ncbi.nlm.nih.gov/). Among them, *Acer saccharum* and *Acer rubrum* were selected as outgroup. The 12 chloroplast genome sequences were aligned using MAFFT v7.310 (Rozewicki, J, et al.,2019) with default parameters. Following sequence alignment, the resulting datasets were trimmed using TrimAl v1.5.0 (Capella-Gutiérrez, S, et al.,2009) with the specified "-automated1" parameter. Phylogenetic analysis was conducted in IQ-TREE v2.4.0 (Minh, B Q, et al.,2020) via the maximum-likelihood (ML) method with 1000 bootstrap replicates, based on the optimal substitution model (TVM+F+I+G4) selected under the Akaike Information Criterion (AIC) using the software’s built-in ModelFinder module (Kalyaanamoorthy, S, et al.,2017). The resulting phylogenetic tree was visualized using the iTOL v7.2.1 web server (Letunic, I and Bork, P,2021).

1. **Results**

3.1 Characteristics of the chloroplast genome

The chloroplast complete genome of *A. pavia* exhibited a typical quadripartite structure, with a total length of 156,394 bp and a GC content of 37.90% (Figure 2). It consisted of a large single-copy (LSC) region of 85,927 bp, a small single-copy (SSC) region of 18,751 bp, and a pair of inverted repeat regions (IRa and IRb), each 25,858 bp in length (Figure 2). The chloroplast genome was sequenced with an average coverage of 4473× (range: 1195×–8596×), which effectively minimized random sequencing errors through consensus calling. More than 95% of the genome regions had a coverage ≥50×, ensuring high confidence in base calling (Figure S1).

A total of 133 genes were annotated in the chloroplast genome of *A. pavia*, including 88 PCGs, 37 transfer RNAs, and 8 ribosomal RNAs. The LSC region contained 69 PCGs and 22 tRNAs, The SSC region contained 12 PCGs and one tRNA. Within the IR regions. Seven PCGs, all rRNAs and seven tRNAs were duplicated. Among these, 10 PCGs (*rps16*, *atpF*, *rpoC1*, *petB*, *petD*, *rpl16*, *rpl2*, *ycf2*, *ndhB*, *ndhA*) contain an intron, and three PCGs (*pafⅠ*, *clpP1*, *ycf1*) contain two introns (Figure S2). All 16 of these genes are cis-splicing genes, which play crucial roles in ensuring the integrity of ribosome biogenesis and protein translation, directly affecting photosynthetic efficiency and plant growth and development (Huo, Y, et al.,2024; Wang, X, et al.,2022). Additionally, the rps12 gene was identified as a trans-splicing gene with three exons (Figure S3), which is essential for the stable operation of basic functions such as photosynthetic systems, energy metabolism, and ribosome assembly (Lee, K, et al.,2019).

We compared and mapped whole chloroplast gene alignments among 10 *Aesculus* species using mVISTA, using the published cp genome of *A. assamica* (NC_056237) as the reference. The results showed that the divergence in the coding regions was greater than that in the noncoding regions (Figure S4). SSRs were analyzed using Misa and CPStools, and the results generated by these two analytical tools were consistent. A total of 71 SSRs were detected in *A. pavia* chloroplast genome, and with mononucleotide repeats being the most abundant (70 out of 71 SSRs).

3.2 Phylogenetic relationship

Phylogenetic analysis was performed using 10 chloroplast genomes from *Aesculus* species, *Acer saccharum* and *Acer rubrum* as outgroup. The ML phylogenetic tree based on the 12 cp genomes revealed two major clades within the genus *Aesculus* (Figure 3), largely consistent with previous studies(Du, Z, et al.,2020). *A. pavia* was closely related to *A.turbinata* and *A.hippocastanum*. Most nodes in the tree exhibited a bootstrap support value of 100%, while only a few nodes in the terminal branches showed slightly lower support values. This indicates that the branching relationships of this phylogenetic tree are generally highly reliable.

**4. Discussion and conclusions**

In this study, the complete chloroplast genome of *A. pavia* was reported for the first time, containing 133 genes, including 88 PCGs, 37 tRNA genes, and 8 rRNA genes. The chloroplast genome of *A. pavia* is similar in size and structure to those of other reported *Aesculus* species, which indicates a relatively conserved chloroplast genome in this genus. We quantified the gene content of the chloroplast genomes from two closely related species, *A. hippocastanum* and *A. turbinata*, and the results demonstrated that the total number of genes was consistent across all three species (Table S1).

Comparative analysis of the inverted repeat (IR) boundaries revealed that the IR region boundaries of *A. pavia* and closely related species exhibited varying degrees of expansion and contraction (Figure S4). In particular, Notably, the lengths of the intergenic spacer regions at the JSB boundary (the junction of IRb and SSC) and the JSA boundary (the junction of IRa and SSC) exhibited distinct expansion. This is one of the core factors contributing to the slight divergence in their total genome lengths (Saina, J K, et al.,2018) and also reflects the evolutionary diversity of chloroplast genomes within the genus *Aesculus*.

Phylogenetic analysis showed that that *A. pavia* was closely related to *A. turbinata* and *A. hippocastanum*. This finding is consistent with previous studies based on RAD sequencing data (Du, Z, et al.,2020). The cp genome sequence of *A. pavia* determined in this study provide important information for phylogenetic and evolutionary studies in *Aesculus*.

**Acknowledgments**

We thank the Shandong Academy of Forestry for providing us with sample. Conceiving and designing, Xuehua Xie; performing and analyzing data, Huihui Lin, Xiangxiao Meng and XiuHong Mao; writing-original draft preparation, Xuehua Xie, Huihui Lin, Xiangxiao Meng, Weqiang Chen, Wei Sun; writing-review and editing, Xuehua Xie, Huihui Lin, Xiangxiao Meng, Huihua Wan, Xuehua Xie; all authors have read and agreed to the published version of the manuscript.

**Author contributions**

CRediT: **Huihui Lin**: Data curation, Formal analysis, Methodology, Software, Writing-original draft, Writing-review & editing; **Xiangxiao Meng** Data curation, Formal analysis, Funding acquisition, Validation, Visualization, Writing-review & editing; **Huihua Wan** Project administration, Resources, Funding acquisition; **XiuHong Mao** Investigation, Resources; **Wei Sun**: Conceptualization, Project administration, Supervision; **Weiqiang Chen**: Project administration, Resources, Funding acquisition; **Xuehua Xie**: Conceptualization, Investigation, Project administration, Resources, Supervision, Writing-original draft, Writing-review & editing.

**Ethical approval**

This study did not involve humans or animals. This study did not require ethical approval or permission to collect samples.

**Disclosure statement**

No potential conflict of interest was reported by the author(s).

**Funding**

This work was supported by the National Natural Science Foundation of China (82304673); the National Key Research and Development Program of China (2023YFC3504104); the scientific and technological innovation project of China Academy of Chinese Medical Sciences (CI2023E002, CI2024E003); the Open Research Fund of Yunnan Characteristic Plant Extraction Laboratory (YKKF2024020); the Fundamental Research Funds for the Central public welfare research institutes (ZXKT22051, ZXKT23029).

**Data availability statement**

The genome sequence data that support the findings of this study are openly available in GenBank of NCBI at https://www.ncbi.nlm.nih.gov under the accession number PX251891. The associated BioProject, SRA, and Bio-Sample numbers are PRJNA1320835, SRR35276515, and SAMN51175175 respectively.

**Figure Legend**

Figure 1. The morphological characteristics of *A.pavia.* (a) The leaves of *A.pavia* . palmate compound leaves, Usually, there are 5 leaflets, and 5 - 7 leaflets on mature trees. The leaf blades are 7.5 - 15 centimeters long, green in summer and golden in autumn. (b) The seeds of *A.pavia.* The fruits are spherical, 2.5 - 5 centimeters in diameter, light brown. There are 1 - 3 seeds in each fruit, and the seeds are poisonous*.* (c) Plant individuals of *A. pavia.* The tree is 5-8 metres tall, with grayish - brown bark. The photos were taken by Xiuhong Mao in Jinan county, Shandong Province, China.

Figure 2. Gene map representing the chloroplast genome of *A. pavia*. As shown, the figure is consisting six of circles from the center to the outside, The innermost circle shows the forward and reverse repeats connected with the red and green arcs, respectively. The second circle and the third circle show the long tandem repeats and short tandem repeats or microsatellite sequences marked with short strips, respectively. The fourth circle exhibits the locations and length of the large single-copy (LSC) regions, small single-copy (SSC) and inverted repeat (IRA and IRB) regions. The fifth and the sixth circle display GC content and the genes’ function categories as shown in different colors. The genes outside the outermost circle are transcribed anticlockwise, while the genes inside are transcribed clockwise. The number in parenthesis after gene name indicates codon usage bias.

Figure 3. Maximum-likelihood (ML) phylogenetic tree based on the complete chloroplast genome sequence of 11 species from the Sapindaceae. Numbers at each node correspond to bootstrap values calculated from 1,000 repetitions. The chloroplast genomes of *A. pavia* in this study were labeled in red and marked with a red star. The sequences used for constructing the phylogenetic tree are as follows: *Acer saccharum* MW067075 (unpublished), *Acer rubrum* MN864509 (unpublished), *A. turbinata* PP809766 (unpublished), *A. hippocastanum* NC_066015 (unpublished), *A. chinensis* var. *chekiangensis* PP809760 (unpublished); *A. chinensis* NC_046788 (Zhang, Z, et al.,2019); *A. chinensis* var. *wilsonii* PP809769 (unpublished); *A. assamica* NC_056237 (unpublished); *A. wangii* NC_035955 (Zheng, W, et al.,2018); *A. tsiangii* PP809765 (unpublished); *A. polyneura* PP809764 (unpublished)*.*

Figure S1. The sequencing depth of the complete chloroplast genome of *A. pavia.* The figure illustrates the sequencing depth distribution across the chloroplast genome, with the minimal, maximal, and average depth of 1195×, 8596×, and 4473×, respectively. The x and y axes represent the nucleotide position and the corresponding sequencing depth, respectively.

Figure S2. The map of the cis-splicing genes, including 16 cis-splicing genes (*rps16*, *atpF*, *rpoC1*, *pafⅠ*, *clpP1*, *petB*, *petD*, *rpl16*, *rpl2*, *ycf2*, *ndhB*, *ndhA*, *ycf1*), the *ndhB*, *ycf2* and *rpl2* were duplicates, 13 of them have one intron and two exons, and three have two introns and three exons.

Figure S3. The map of the trans-splicing gene in the *A. pavia* chloroplast genome.

Figure S4. Comparison of the ten Aesculus species cp genomes using the mVISTA alignment program, with *A. assamica* (NC_056237) as a reference.

Figure S5. Chloroplast genome boundary comparisons of three *Aesculus* species. This figure shows the boundary regions (JLB: IRb/LSC; JSB: IRb/SSC; JSA: IRa/SSC; JLA: IRa/LSC) in the chloroplast genomes of *A. hippocastanum* (NC_066015), *A. turbinata* (PP809766), and *A. pavia* (PX251891). Total genome lengths (below species names) and lengths of LSC/IR/SSC regions (adjacent to segments) are marked; genes spanning boundaries (e.g., *rpl2*, *ycf1*) and their cross-boundary lengths (in bp) are annotated near each boundary.

Table S1. Comparison of the number of genes in the chloroplast genomes of A. pavia, *A. hippocastanum*, and *A. turbinate*.

**Reference**

Beier S, Thiel T, Münch T, et al. MISA-web: a web server for microsatellite prediction[J]. Bioinformatics, 2017,33(16):2583-2585.

Capella-Gutiérrez S, Silla-Martínez J M, Gabaldón T. trimAl: a tool for automated alignment trimming in large-scale phylogenetic analyses[J]. Bioinformatics, 2009,25(15):1972-1973.

Chen S. fastp 1.0: An ultra-fast all-round tool for FASTQ data quality control and preprocessing[J]. iMeta, 2025,4(5):e70078.

Du Z, Harris A J, Xiang Q J. Phylogenomics, co-evolution of ecological niche and morphology, and historical biogeography of buckeyes, horsechestnuts, and their relatives (Hippocastaneae, Sapindaceae) and the value of RAD-Seq for deep evolutionary inferences back to the Late Cretaceous[J]. Molecular Phylogenetics and Evolution, 2020,145:106726.

Guo C, He Y, Zeng X, et al. Chloroplast DNA reveals genetic population structure in *Sinomenium acutum* in subtropical China[J]. Chinese Herbal Medicines, 2023,15(2):278-283.

Huang L, Yu H, Wang Z, et al. CPStools: A package for analyzing chloroplast genome sequences[J]. iMetaOmics, 2024,1(2):e25.

Huo Y, Cheng M, Tang M, et al. GhCTSF1, a short PPR protein with a conserved role in chloroplast development and photosynthesis, participates in intron splicing of rpoC1 and ycf3-2 transcripts in cotton[J]. Plant Commun, 2024,5(6):100858.

Jin J, Yu W, Yang J, et al. GetOrganelle: a fast and versatile toolkit for accurate de novo assembly of organelle genomes[J]. Genome Biology, 2020,21(1):241.

Kalyaanamoorthy S, Minh B Q, Wong T K F, et al. ModelFinder: fast model selection for accurate phylogenetic estimates[J]. Nature Methods, 2017,14(6):587-589.

Lee K, Park S J, Colas Des Francs-Small C, et al. The coordinated action of PPR4 and EMB2654 on each intron half mediates trans-splicing of rps12 transcripts in plant chloroplasts[J]. Plant J, 2019,100(6):1193-1207.

Letunic I, Bork P. Interactive Tree Of Life (iTOL) v5: an online tool for phylogenetic tree display and annotation[J]. Nucleic Acids Res, 2021,49(W1):W293-W296.

Liu S, Ni Y, Li J, et al. CPGView: A package for visualizing detailed chloroplast genome structures[J]. Mol Ecol Resour, 2023,23(3):694-704.

Liu Z, Zhang J, Zhou Y, et al. The complete chloroplast genome of *Aesculus chinensis* var. *wilsonii*[J]. Mitochondrial DNA B Resour, 2020,5(3):2547-2549.

Minh B Q, Schmidt H A, Chernomor O, et al. IQ-TREE 2: New Models and Efficient Methods for Phylogenetic Inference in the Genomic Era[J]. Mol Biol Evol, 2020,37(5):1530-1534.

Modliszewski J L, Thomas D T, Fan C, et al. Ancestral chloroplast polymorphism and historical secondary contact in a broad hybrid zone of *Aesculus* (Sapindaceae)[J]. Am J Bot, 2006,93(3):377-388.

Rozewicki J, Li S, Amada K M, et al. MAFFT-DASH: integrated protein sequence and structural alignment[J]. Nucleic Acids Research, 2019,47(W1):W5-W10.

Saina J K, Li Z, Gichira A W, et al. The Complete Chloroplast Genome Sequence of Tree of Heaven (Ailanthus altissima (Mill.) (Sapindales: Simaroubaceae), an Important Pantropical Tree[J]. Int J Mol Sci, 2018,19(4).

Shi L, Chen H, Jiang M, et al. CPGAVAS2, an integrated plastome sequence annotator and analyzer[J]. Nucleic Acids Res, 2019,47(W1):W65-W73.

Sirtori C R. Aescin: pharmacology, pharmacokinetics and therapeutic profile[J]. Pharmacol Res, 2001,44(3):183-193.

Sun Z, Zhang M, Wu Y, et al. Bioactive saponins from the fruits of *Aesculus pavia* L.[J]. Fitoterapia, 2011,82(7):1106-1109.

Tillich M, Lehwark P, Pellizzer T, et al. GeSeq - versatile and accurate annotation of organelle genomes[J]. Nucleic Acids Research, 2017,45(W1):W6-W11.

Wang X, Wang J, Li S, et al. An overview of RNA splicing and functioning of splicing factors in land plant chloroplasts[J]. RNA Biol, 2022,19(1):897-907.

Zhang Z, Chen Y, Jiang X, et al. The complete chloroplast genome of *Aesculus chinensis*[J]. Mitochondrial DNA Part B, 2019,4(1):1955-1956.

Zhang Z, Li S. Cytotoxic triterpenoid saponins from the fruits of *Aesculus pavia* L.[J]. Phytochemistry, 2007,68(15):2075-2086.

Zhang Z, Li S, Zhang S, et al. Triterpenoid saponins from the fruits of Aesculus pavia[J]. Phytochemistry, 2006,67(8):784-794.

Zheng W, Wang W, Harris A J, et al. The complete chloroplast genome of vulnerable *Aesculus wangii* (Sapindaceae), a narrowly endemic tree in Yunnan, China[J]. Conservation Genetics Resources, 2018,10(3):335-338.
